# Authors’ reply: Geographic resolution of surveillance data and influenza prevention in large countries

**DOI:** 10.2807/1560-7917.ES.2017.22.40.17-00671

**Published:** 2017-10-05

**Authors:** Saverio Caini, Wladimir J Alonso, Clotilde El-Guerche Séblain, François Schellevis, John Paget

**Affiliations:** 1 Netherlands Institute for Health Services Research (NIVEL), Utrecht, The Netherlands.; 2 Origem Scientifica, São Paulo, Brasil.; 3 Sanofi Pasteur, Lyon, France.; 4 Department of General Practice and Elderly Care Medicine, EMGO Institute for Health and Care research, VU University Medical Center, Amsterdam, The Netherlands.

**Keywords:** Influenza, surveillance, Europe, Russian Federation

**To the editor:** We would like to thank GY Shin and R Manuel for the attention they have given to our article. We believe that the point raised in their letter is valid and, in fact, we pointed out this important limitation in the paper ourselves. The Russian Federation has a very large population that is unevenly distributed over its vast territory, with populous cities surrounded by areas with very low population density. Because of this, we agree that representing the Russian Federation with a single geographical point (be it Moscow, as we did in our paper, Saint Petersburg, where a second National Influenza Centre (NIC) is situated, or the country centroid) is far less than optimal. 

The authors suggest that complex spatiotemporal patterns of influenza may exist within the Russian Federation, which could therefore “*be an ITZ on its own*” or even “*encompass more than one ITZ*”. These are sensible hypotheses that would be worth examining in detail; unfortunately, however, this is currently not possible using the FluNet database, which contains influenza surveillance data for individual countries but not for their subnational entities (e.g. administrative divisions such as the eight federal districts of the Russian Federation). 

Large countries may encompass areas with different seasonality of influenza epidemics (due to diversity in climates and other factors), and may therefore greatly benefit of tailored recommendations regarding the influenza vaccine composition and optimal time of administration (e.g. China [1], India [2], Brazil [3] or Mexico [4]). Accordingly, we agree with the suggestion that one should establish more than one NIC in large countries, and we believe that the World Health Organization could further assist the efforts in this area by making influenza surveillance data available at a finer geographical resolution for large countries in the world.
